# Incorporating personal narratives in positive psychology interventions to manage chronic pain

**DOI:** 10.3389/fpain.2023.1253310

**Published:** 2023-10-06

**Authors:** Emmanouil Georgiadis, Mark I. Johnson

**Affiliations:** ^1^ School of Social Sciences and Humanities, University of Suffolk, Ipswich, United Kingdom; ^2^ Centre for Pain Research, School of Health, Leeds Beckett University, Leeds, United Kingdom

**Keywords:** personal narrative, chronic pain, positive psychology, agency, healthcare

## Abstract

In this perspective paper, we argue for incorporating personal narratives in positive psychology interventions for chronic pain. Narratives refer to the telling and retelling of events. Narratives detail accounts of events and provide rich, in-depth information on human interactions, relationships, and perspectives. As such, narratives have been used to understand people’s experiences with pain and pain coping mechanisms—as well as to facilitate therapeutic outcomes. Furthermore, narrative research has shown that narration can help restore and promote relief, calm, hope, self-awareness, and self-understanding in chronic pain sufferers. Positive psychology interventions have been successful in improving the lives of people living with chronic pain, but these psychology interventions do not typically incorporate personal narratives. Still, narrative, and positive psychology scholarship foci overlap, as both aim to enhance people’s quality of life, happiness, and well-being, and to promote the understanding of psychosocial strengths and resources. In this article, we provide a rationale for incorporating personal narratives as an agentic form of positive psychology intervention. To that aim, we outline areas of convergence between positive psychology and narrative research and show how combining positive psychology exercises and narration can have additive benefits for pain sufferers. We also show how integrating narration in positive psychology intervention research can have advantages for healthcare research and policy.

## Introduction

Chronic (persistent or long-standing) pain is defined by the World Health Organisation and by the International Association for the Study of Pain as pain persisting or recurring for longer than 3 months (1, 2). It is estimated to affect more than 20% of the adult population contributing significantly to the global burden of disease with significant impact on mortality and disability across human societies worldwide (3). Chronic pain is associated with reduced functional capacity, low sense of wellbeing, impaired social interactions, medication over-prescription, illegal substance use and abuse, mood disorders, suicidal ideation and suicide attempts; this results in low quality of life (4).

It is widely acknowledged that a multidisciplinary person-centred biopsychosocial approach using pharmacotherapy, restorative therapies, behavioural treatments, complementary and integrative therapies, and self-care is optimal to manage chronic pain (5). It is recommended that safe, less invasive treatments should be indicated first (5–8), yet biomedical interventions (i.e., surgical and pharmacological) still dominate clinical practice, despite evidence of unfavourable risk benefit profiles (5, 9, 10).

The biopsychosocial model of pain, evolved from Melzack and Wall’s theory of pain (11) and Engel’s new model for medicine (12) over 40 years ago [for a historical account see (13)]. The biopsychosocial model describes a dynamic interaction of biological (e.g., tissue injury, genetics, neurobiology, sex differences, physical health conditions), psychological (e.g., thoughts, beliefs, attitudes, emotions, coping patterns), and social (e.g., poverty, access to welfare, stigma, discrimination) influences to explain between and within person variability in pain experience, including cognitive appraisal of symptoms. Biopsychological approaches to the management of pain are incorporated within an interdisciplinary model of care, emphasizing holistic patient-centred strategies, that combine pharmacotherapy, physical therapy, cognitive-behavioural counselling and other psychotherapeutic modalities, progressive muscle relaxation, risk-reduction training, biofeedback training, social support groups and networking (14, 15).

In this article, we explore the psychological aspect of the biopsychosocial model of pain and advocate the inclusion of personal narratives as part of psychological interventions used in chronic pain management. Specifically, we advocate a positive psychology approach to personal narration and outline areas of convergence between positive psychology and narrative research. We show how combining positive psychology techniques and narration can have synergistic benefits for pain sufferers, and how narration can have advantages for healthcare research and policy.

## Personal narratives and healthcare

Narratives are personal stories through which humans tell and retell events (16). Narratives describe events but also become part of the events. Narratives merge with the narrator’s reality but may not be accurate depictions of events and may or may not be linear in time and place. Narratives provide rich, in-depth insights on human experiences, interactions, relationships, and behaviours; they are a tool for sense-making and meaning-making (17).

In recent years narrative literature in healthcare has been used to gain insight to the attitudes of individuals about treatments, therapy, barriers preventing treatment, and unaddressed means for enhancing overall quality of life (18). Research evidence suggests short/medium term benefit of using narrative as part of therapy and there are calls for narrative to be included in health care policies (19, 20).

Therapeutically, personal narratives have been shown to support personal values, identity formation, positive emotions, resilience and purpose (21). For example, encountering a negative event such as a life-threatening diagnosis, can turn into a positive story of experiencing redemption via personal courage and realisation of deeper meaning and a new purpose in life. This can increase self-worth and resilience (22, 23).

## Personal narratives and pain management

Pain is a private, complex, organic, multidimensional experience that is idiosyncratic in nature (24, 25). People living with persistent pain experience difficulties constructing meaningful explanations for their pain and suffering (26, 27). People struggle to articulate their personal pain story in a way that is understood, heard, or taken seriously by others, including health care professionals (28–31). In clinical consultation, patients are often forced to express pain through assessment tools that collapse the multi-faceted nature of pain into simplified generalisations or into individual, disconnected, items that fragment pain experience (31). Being unable to express pain through personal narrative results in decontextualise care that is detrimental to health and well-being (32).

Human social groups bond through gossiping and storytelling (33, 34). The act of storytelling personally significant events and contemplating towards lessons learnt and future actions, confers benefit for people living with chronic pain (35). Evidence suggests that personal narrations can improve wellbeing when linked to positive expectations of longer-term recovery, such as relief from pain and improvement in functioning (36). An appraisal of the value of personal story for people living with chronic pain by Hovey et al. suggested that narratives help people interpret their pain and their new lived experience with pain (37). Personal narratives with optimistic content have been associated with beneficial outcomes including enhanced psychological states such as acceptance of areas beyond personal control, positive reinforcement, progress to new achievements, new personal meaning, and motivation to continue exercise, which improve health, well-being and quality of life (35, 38).

## Positive psychology for pain management

Equally, recent findings support the ability of positive psychology interventions to improve quality of life and the severity and impact of pain. The operational mechanism of positive psychology intervention is relatively simple. During pain, negative emotions may exist to instigate behaviours to protect tissue from actual or potential harm (39). However, adhering to action preventing negative emotions may exacerbate negative thinking and destructive behavioural patterns (i.e., heightened worry and avoidance of movement), with pain becoming chronic and increasingly devastating (40). Engaging in frequent positive psychology exercises seems to offset the links between pain and negative emotions (e.g., fear, anxiety and sadness) and cognitions (e.g., rumination, worry, helplessness and catastrophising), eliciting positive sentiments instead (41).

A systematic review of 16 RCTs by Braunwalder et al. (42) provides tentative evidence that positive psychology interventions, delivered as online self-help or guided face-to-face interventions are efficacious to alleviate chronic pain. Thus, simple, regular, positive psychology exercises, may help to reduce the severity and impact of chronic pain. Examples of positive exercises that encourage strengthening and enjoyment of social connections and human relationships to improve perceived pain, emotional states and physical function include performing good deeds to other people, reflecting on blessings, appreciating life circumstances, feeling grateful, and pursuing meaningful and significant goals in daily living (42, 43).

Positive psychology interventions aiming to alleviate chronic pain and its consequences in daily living are usually delivered online and asynchronously (without the presence of a specialist) providing a cost-effective technique (42). Successful implementation requires participants to dedicate personal time and effort to execute these exercises repetitively, based on self-selected pace and personal understanding.

Practitioners define the nature of the intervention to meet desired outcomes (i.e., improved social connection), although sometimes participants are given an opportunity to select which type of positive psychology technique they wish to use (44). For example, in a study by Muller et al. (43) participants had the option to engage in one or more of four types of positive psychology exercises; relationships, kindness, gratitude, optimism. Through this methodology design, autonomy of choosing a positive psychology exercise seems to be enhanced. Positive psychology intervention seems contrary to the concept of “agency” as defined by Seligman (45). Seligman (45) defines agency as the power behind each person’s belief that can change the world, or more precisely here, change an individual’s sense making of the world. Agency relates to the efficacy to act, based on self-defined criteria, with optimism and inspiration being integral to the will to control one’s own fate irrespective of life’s adversities (45). Clearly, agency requires personal freedom to implement self-defined priorities in one’s life. Such degree of freedom seems to be missing from existing positive psychology techniques aiming to support wellbeing indices in chronic pain. This is based on the philosophical, epistemological and practical background of positive psychology, which can be significantly enriched with an emphasis on agency and personal responsibility (46).

## Personal narratives in positive psychology interventions to manage pain

We advocate the use of personal narratives as an innovative positive psychology technique through protocols that support personal agency and autonomy for the participants. Positive psychology interventions provide encouraging results when used for people living with chronic pain (42), and this is achieved by improving components of the Positive emotions, Engagement, valued/supported Relationships, Meaningfulness and Achievement (PERMA) model (47). Personal narratives empower individuals with greater ownership of the direction, pace and content of sense making through exploration of *personal* memories, that make autobiographical sense, through the realisation of dispositional traits and how these may adapt under current circumstances. Hence, personal narratives define identity in various ways but most importantly, via the capacity to keep a unique agentic and personally-defined narrative, using dispositional traits under certain socio-cognitive, developmental, and environmental requests (23). Applying personal narratives to a previously challenging or traumatising event improves quality of life and daily functioning (48, 49). However, the use of personal narrative has not been integrated with positive psychology techniques that have shown capacity to support positive emotions by improving engagement, purpose, achievement, and human relationships.

One way to combine agency and positive psychology techniques is starting with the examination of personal beliefs and values through the use of the Values In Action (VIA) Inventory of Strengths instrument (VIA-IS instrument, http://www.viacharacter.org/ (50), updated in 2019 to the VIA Inventory of Strengths-Revised [VIA-IS-R (51)]). The original VIA-IS was a 240-item questionnaire and the VIA-IS-R consists of 196 items (52) measuring 24 key character strengths (e.g., creativity, bravery, teamwork) based on 6 distinct virtues (wisdom and knowledge, courage, humanity, justice, temperance, transcendence).

The VIA-IS instrument is used to profile personal strengths to aid integration of these character strengths into daily living, by setting goals and acting purposefully to enhance well-being, improve self-acceptance, and boost life-satisfaction (53). The results of the VIA-IS questionnaire are applied to a three-step process, Aware-Explore-Apply, to create a framework to navigate how identified strengths can improve happiness, boost relationships, and contribute to a better outlook for the future (54). After recognising preferred virtues and personal strengths, individuals are invited to narrate their personal story based on significant past events, and by looking at their present and future lives, with special reference to experiences that contain those preferred virtues and strengths. We provide a possible intervention schedule in Table 1.

**Table 1:** Stages and meetings of a positive psychology with personal narrative intervention.

Suggested Intervention Protocol. Meetings can be in person and/or online via teleconferencing platforms	
Meeting 1 (Counsellor)	30 minutes meeting with a specialist counsellor to discuss the stages of the suggested protocol starting with stage 1 (VIA-IS Questionnaire). The goal of the session is to respond to the question “*What will I get from the VIA-IS questionnaire?”*
Stage 1 (Independent)	Completion of VIA-IS Questionnaire (https://www.viacharacter.org/) at the person’s chosen time and place
Meeting 2 (Counsellor)	30 minutes meeting with a specialist to discuss Stage 2 of the life-story interview protocol and how it works. The goal of the session is to respond to the question “*What will I get from the life-story interview?”*
Stage 2 (Independent)	The life-story interview. The goal of this stage is for the person to appreciate significant events, challenges, and accomplishments in life. Allow up to 15 days for the person to complete Stage 2 at their chosen time and place.
Meeting 3 (Counsellor)	60 minutes meeting with a specialist to go through the three-step Aware-Explore-Apply process to explore the results of the VIA-IS questionnaire. Realisations from the life-story interview are discussed and preparatory work is undertaken for the design of the final stage of the life-story interview (future life). There are 2 objectives; (i) to be aware and explore ways personal strengths have been shaping previous significant events and accomplishments in life highlighted in Stage 2 via the life-story interview, and (ii) realise and decide on which personal strengths to activate to derive personal fulfilment, success, and control in daily living. The goal of the session is to respond to the question: “*How can I use my personal strengths to energise my life?”*
Stage 3 (Independent)	Creation of the future life. The goal of the stage is for the person to clarify how stages 1 and 2 can support a personally fulfilling life by responding to the question “*How can I shape my future?”*. Allow up to 15 days for the person to complete Stage 3 at their chosen time and place.
Meeting 4 (Counsellor)	60 minutes meeting with a specialist to go through the whole experience of the intervention protocol and shape personal behaviours (e.g., future plans, actions, realisations). The goal of the session is to respond to the question “*What are the my realisations from this experience?*”
Meetings 5, 6 and 7 (Counsellor)	30 to 60 minutes monthly or bi-monthly meetings with a specialist to check progress and support the person activate their new plan. The goal of each session is to respond to the question “*How am I progressing based on the goals I have set?*”

### The life story interview

We advocate the use of a “life story interview” in the form of a personal written narrative, to facilitate this process (55). The life story interview relates to a metaphor based on which major life events are contributing to the main chapters of one’s life. After defining those, the individual considers the high, the low and the turning point, as well as the positive experience and wisdom that derives from each one of those important life events. The ultimate phase relates to the individual’s aspiration regarding the next chapter of their life story, personal hopes and future life project that is expected to be in accordance with the report of the already completed VIA-IS questionnaire. Personal agency is manifested here on three levels:

1. The exploration of personal values and strengths,
2. The availability of ample time and self-selected pace to complete one’s life story interview, and
3. The future life project and its numerous options for the individual.

Based on significant events and challenges, and through the comparison of already performed behaviours versus personally valued actions, each individual has an increased chance to realise areas of reduced control and opportunities for personal growth. It also allows for contemplating continuity of personal identity based on valued activities and actions (53).

With an aim to reverse the negative experiences of chronic pain, the goal of the life story narrative technique is to create personal and agentic experiences of redemption leading to a revitalised connection to personal meaning, engaging behaviours and flow, synergetic relationships, personal activation, optimism, and increased resilience (see example in Table 2). Similarly to other positive psychology interventions, this technique can be implemented repetitively (thematically and until long-term resilience is established), online and in an asynchronous way, providing a low-cost alternative to expensive pain treatments.

**Table 2:** Example of how positive psychology with personal narrative can change a person’s outlook.

Negative narrative of an individual with persistent pain
The pain is always there. It’s a dull ache that never goes away, and it's always worse in the morning. It's hard to get out of bed, and it's even harder to do anything productive. I used to be so active, but now I just sit around all day. I've lost touch with my friends, and I'm starting to feel like I'm losing my mind. I've been to the doctor, but they can't find anything wrong with me.
They say it's just chronic pain and there's nothing they can do. I'm starting to think they're right. I'm just going to have to live with this pain for the rest of my life. I'm starting to detach from my social connections. I don't want to see my friends because I don't want them to see me like this. I'm tired of being the friend who's always in pain. I'm tired of being the friend who can't do anything fun. I'm just tired. I'm starting to feel like I'm all alone in this. I don't know anyone else who understands what I'm going through. I feel like I'm losing my grip on reality. I'm starting to wonder if this pain is ever going to end. I'm scared. I don't know what to do. I just want the pain to stop.
Positive narrative of an individual with persistent pain
I was diagnosed with chronic pain when I was 45 years old. It was a devastating diagnosis, and I didn't know how I was going to cope. I had always been an active person, and I loved to play sports and go hiking. But now, I was in constant pain, and I couldn't do the things I loved. At first, I was really depressed. I felt like my life was over. But then through a suggested intervention, I started to realise and apply my strengths in my daily living. This allowed me to take important decisions about my future and my options to shape my life according to my preferences.
I'm not afraid of a challenge. I'm also a positive person, and I always try to find the silver lining. So, I decided that I wasn't going to let chronic pain define me. I was going to find a way to live a happy and fulfilling life, even with pain. It hasn't been easy. There have been times when I've wanted to give up. But I've always found a way to keep going. I've learned to manage my pain, and I've found new ways to be active. I've also found a great support system of friends and family who have helped me through the tough times. I'm not cured, and I may have chronic pain for many more years, but I've learned to live much more positively with it, and I'm not going to let it stop me from living a fulfilling life. Here are some of the personal strengths that have helped me cope with chronic pain; **resilience**—I don't give up easily, **optimism**—I try to find the silver lining, even in the darkest of times, **adaptability**—I've learned to adapt to my pain, and I've found new ways to be active and enjoy life, and **social connection**—I have a great support system of friends and family who have helped me through the tough times.

## Narratives in healthcare pain policy development

The National Institute for Health and Care Excellence (NICE) guidelines on chronic pain highlight the importance of person-centred assessment and management (7). The NICE guidelines emphasise the need to support individuals’ control and autonomy over their condition and social/psychological circumstances, while using an active exploration of personal strengths to improve management of chronic pain. Even though these guidelines propose a holistic approach to the management of chronic pain (i.e., psychological, social, and pharmacological) they endorse psychological approaches that are relatively expensive and time consuming (i.e., Acceptance and Commitment Therapy (ACT) and Cognitive Behavioural Therapy (CBT)). Positive psychology with personal narrative aligns with a whole-person-centred biopsychosocial paradigm of care and empowers people to take an active role in their healing journey to foster adaptive, resilient and autonomous lifestyles against adversities linked to chronic pain (19).

The cost of interventions using personal narratives within a framework of positive psychology is likely to be competitive compared with existing psychological approaches e.g., four hours to learn how to self-administer a positive psychology approach to personal narration (Table 1) compared with 6–20, one hour clinically supervised sessions of CBT. For these reasons we call for evaluations of positive psychology with personal narratives to provide robust research evidence to enable health policy and clinical decision makers to judge the cost-benefit-safety profile.

## Conclusion

A positive psychology approach to personal narration sits within a whole-person paradigm capturing a holistic and biopsychosocial multidimensional method of care (56). Following our appraisal of extant literature, we conclude that integration of personal narratives within a framework of positive psychology offers an innovative agentic technique to assist the psychological states for people living with chronic pain in clinical and non-clinical settings. A recent systematic review provides tentative evidence that positive psychology interventions are efficacious for chronic pain. There is a paucity of research on which to judge the efficacy of integrating personal narrative into positive psychology interventions. Thus, we recommend a scoping review and evidence gap map to inform the direction of future research. We hope that this article stimulates further debate on the topic.

## Data Availability

The original contributions presented in the study are included in the article/Supplementary Material, further inquiries can be directed to the corresponding author.
